# “Cyclopropylidene Effect” in the 1,3-Dipolar Cycloaddition of Nitrones to Alkylidene Cyclopropanes: A Computational Rationalization

**DOI:** 10.1021/acs.jpca.1c02204

**Published:** 2021-04-30

**Authors:** Lorenzo Briccolani-Bandini, Marco Pagliai, Franca M. Cordero, Alberto Brandi, Gianni Cardini

**Affiliations:** Dipartimento di Chimica ”Ugo Schiff”, Università degli Studi di Firenze, Via della Lastruccia 3-13, 50019 Sesto Fiorentino, Firenze, Italy

## Abstract

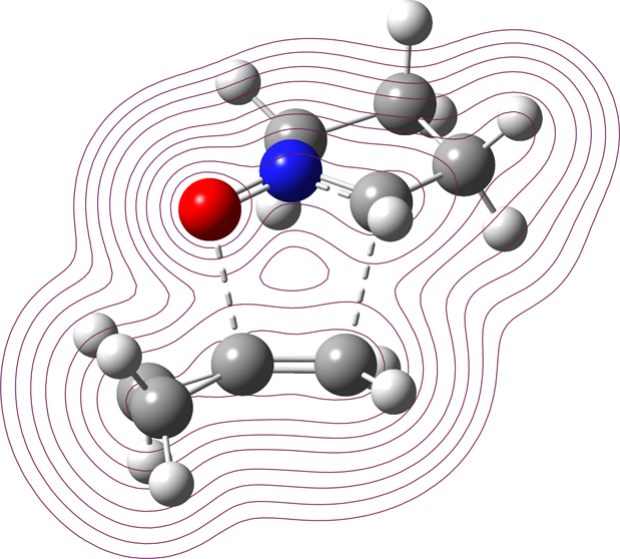

The regioselectivity in the 1,3-dipolar cycloaddition (1,3-DC) between five-membered cyclic nitrone and methylenecyclopropane (MCP) has been studied through density functional theory (DFT) calculations. The computational study of 1,3-DC with different 1-alkyl- (or 1,1-dialkyl)-substituted alkenes and the comparison with MCP have evidenced that the electrostatic interaction has a central role in the regioselectivity of the reactions. It has been observed that the electronic effect of the substituent (donor or attractor groups) determines the polarization of the alkene double bond and the reaction mechanism, consequently determining the interaction with nitrones and favoring an orientation between this moiety and the dipolarophile.

## Introduction

**Scheme 1:**
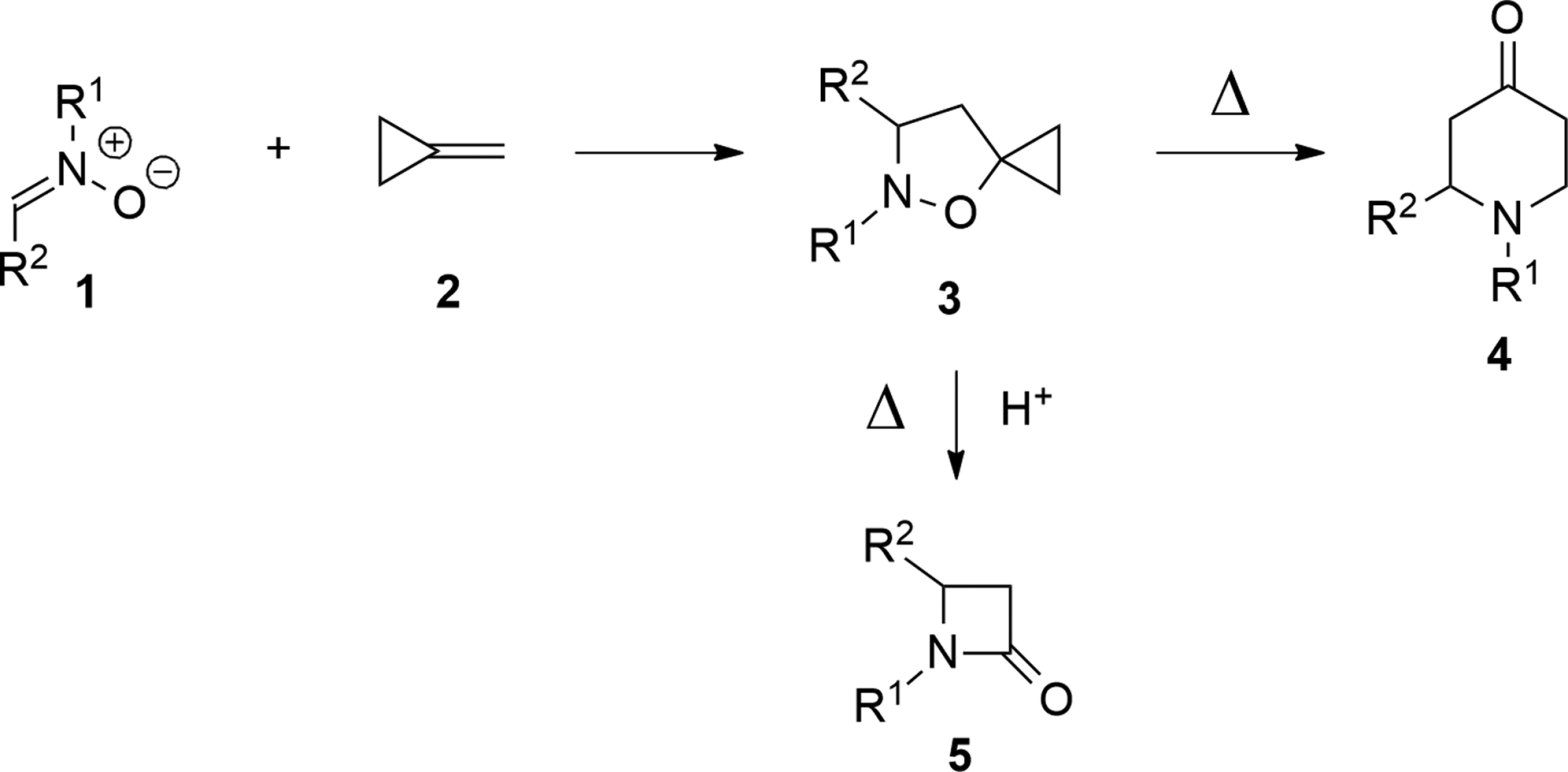
Synthesis and Transformations of 5-Spirocyclopropane Isoxazolidines **3**

The value of thermal rearrangement of 5-spirocyclopropane isoxazolidines **3** (Brandi-Guarna rearrangement, see Scheme 1)^1,2^ for the synthesis of monocyclic and polycyclic heterocycles containing a tetrahydropyridinone ring 4 has been largely proved in recent years.^3,4^ The rearrangement is made possible by the presence in the molecule of a strained oxyspirocyclopropane moiety, where the oxygen is linked to a nitrogen with a bond that is easily cleaved under thermal activation.^5,6^ Another important synthetic application of 5-spirocyclopropane isoxazolidines **3** is the synthesis of β-lactams **5** by thermal fragmentation under acidic conditions.^7−9^ Isoxazolidines **3** find their origin in a 1,3-dipolar cycloaddition (1,3-DC) of nitrones **1** with methylenecyclopropane (MCP, **2**).^10−12^

**Scheme 2:**
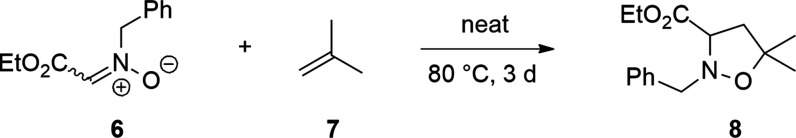
1,3-DC of Nitrone **6** with Isobutene (**7**)

**Scheme 3:**
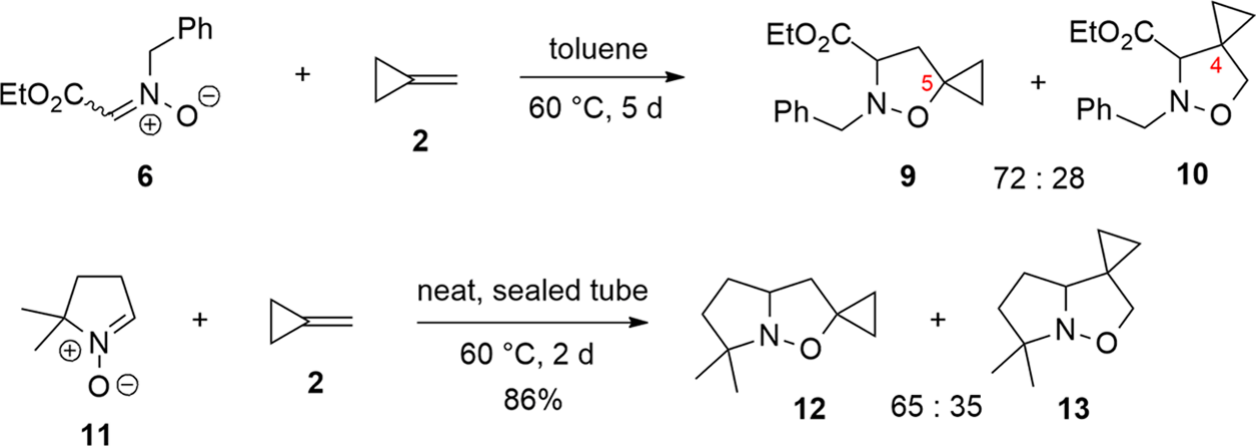
1,3-DC of Nitrones **6** and **11** with MCP (**2**)

MCP is a rather volatile alkene, commercially available, that, despite its strained nature, results rather sluggish in its reactivity with nitrones. The cycloaddition process requires heating above 60 °C. The same occurs in the cycloaddition of nitrones with 1-alkyl- or 1,1-dialkyl-substituted alkenes that is even more slow.^13^ This fact is not surprising knowing the nature of nitrones as electron neutral dipoles (Sustmann’s classification)^14^ and of dipolarophiles missing any activating electron-withdrawing group. However, the similarities of MCP with these alkenes end at this point, because regarding regioselectivity, the behavior of MCP is rather different from a normal 1-alkyl- or 1,1-dialkyl-substituted alkene. Indeed, although a dipolarophile like isobutene (**7**), which is the alkene with the highest similarity to MCP, reacts with nitrone **6** to give exclusively one adduct, i.e., the 5,5-disubstituted isoxazolidine **8** (Scheme 2),^15^ MCP generally affords a mixture of regioisomers, where the 5-spirocyclopropane isoxazolidine is the major, but not the exclusive cycloadduct, as shown in the examples of Scheme 3.^16,17^

**Scheme 4:**
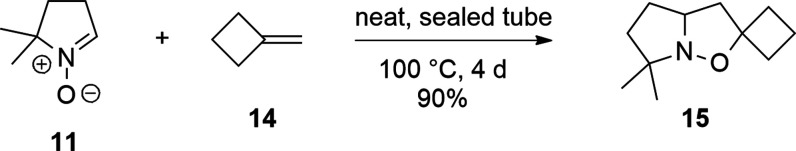
1,3-DC of Nitrone **11** with MCB (**14**)

The awkwardness of this result with regard to the application of this process as a function of the successive thermal rearrangement or β-lactam synthesis is rather evident. In fact, regioisomeric 4-spirocyclopropane isoxazolidines, like **10** and **13** (Scheme 3), lacking the oxyspirocyclopropane moiety, are not able to undergo any useful rearrangement. However, the unexpected lack of regioselectivity in 1,3-DC of MCP raises several questions about the nature of its double bond and its resemblance with a simple alkene. More concerns are added if we consider the cycloaddition to another similar alkene, methylenecyclobutane (MCB, **14**) (Scheme 4), where a cyclobutane replaces the cyclopropane ring.^18^

**Scheme 5:**
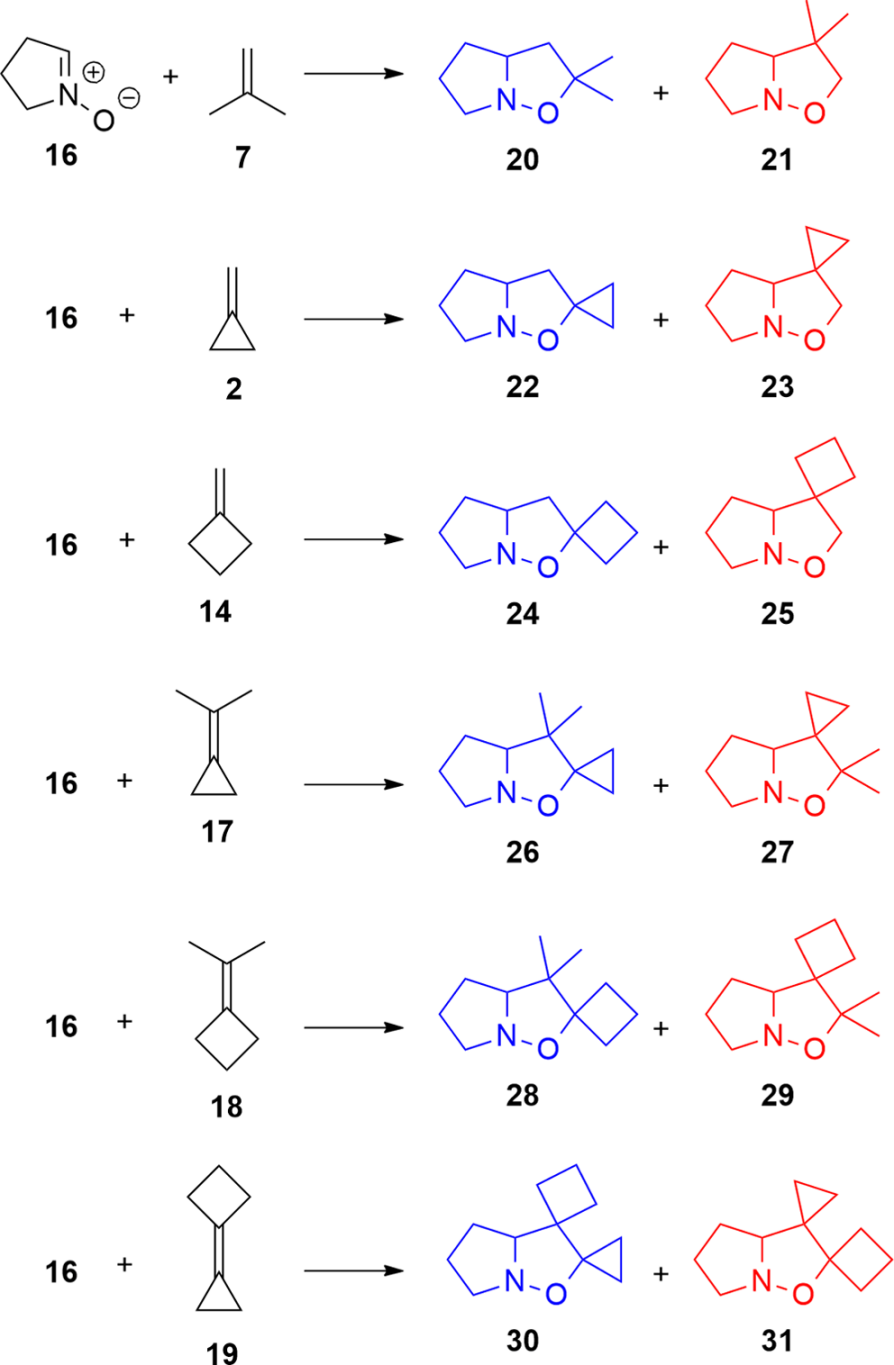
Reactions Studied Through DFT Calculations at the PBE0/6-311G(d,p)-GD3 Level of Theory

If we assume that the strain energy of the ring has a role in the regioselectivity of the cycloaddition, a similar result for MCP and MCB should be expected. Indeed, the cycloaddition of **11** with MCB affords exclusively regioisomer **15**, analogously to the regioselectivity obtained with isobutene (**7**). These data make clear that there must be a “cyclopropylidene effect” in the 1,3-DC with nitrones to justify the observed lack of high regioselectivity. The 1,3-DC reaction mechanism has been computationally investigated with density functional theory (DFT) and post-HF methods: it consists of a concerted, often asynchronous, pericyclic cycloaddition mechanism.^19^ To elucidate this possible cyclopropylidene effect on the regioselectivity of the 1,3-DC of MCP with nitrones, DFT calculations have been carried out on the systems summarized in Scheme 5. The choice of a five-membered cyclic nitrone like **16** for the study is justified by the copious literature available for the cycloadditions of cyclopropylidene dipolarophiles with this class of nitrones.^17,20^ In addition, nitrone **16** featuring a defined configuration eliminates the *E*,*Z*-configuration variable that should be considered in the case of acyclic nitrones. Seven substituted dipolarophiles have been analyzed to investigate the electronic effect of the substituents: isobutene (**7**), methylenecyclopropane (MCP, **2**), methylenecyclobutane (MCB, **14**), isopropylidenecyclopropane (ICP, **17**), isopropylidenecyclobutane (ICB, **18**), and cyclobutylidenecyclopropane (CPCB, **19**) (Scheme 5).

## Computational Details

The reaction mechanisms have been characterized by performing DFT calculations at the PBE0/6-311++G(d,p)^21−24^ level of theory including Grimme’s empirical dispersion (GD3) with the Gaussian suite of programs.^25,26^ Transition states (TS), prereactive minima, and products have been located through geometry optimization calculations with very tight convergence criteria. The QST2^27^ or QST3^28^ algorithms have been adopted to determine the transition states. All of the Hessian eigenvalues are positive for the prereactive minima and products, while only one negative eigenvalue has been obtained for the TS. The eigenvector, corresponding to the negative eigenvalue, describes the displacements along the formed C–C and C–O bonds, providing insights into the concerted reaction mechanism. Different analyses have been carried out to describe the behavior of alkenes with nitrone **16** and to understand the cyclopropylidene effect. The characterization of the stationary points allows us to determine the energy gaps between the TS and reagents or products, to assess any possible kinetic control of the reaction and the balance between the products. The electronic effects of the alkyl substituent on the alkenes have been investigated by the value and the orientation of the dipole moment on the reagents and through the determination of the electrostatic potential (ESP) atomic charges.^29^ To further support the electronic structure analysis, comparisons between the ESP atomic charges and those obtained through the CM5^30^ and NPA^31,32^ methods have been reported in Tables S1–S7 in the Supporting Information. The kinetic constants of the reactions have been calculated using the Eyring transition state theory.^33−35^ The results allowed a comparison of the kinetic parameters for the two different orientations of the alkenes for each reaction in Scheme 5 and to explain the different experimental yields. For the studied systems, the expression of the kinetic constant *k*(*T*) in the Eyring approximation is simplified, treating the reagents in the prereactive minimum as a single molecular complex, and results
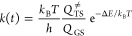
1where *k*_B_ is the Boltzmann constant, *T* is the temperature, *h* is the Planck’s constant, and Δ*E* is the energy gap (zero-point energy included). The two functions are the product of vibrational and rotational partition functions for the transition state *Q*_TS_^≠^ and ground state *Q*_GS_. The kinetic constants have been calculated for the experimental reaction temperature range between 300 and 400 K.

## Results and Discussion

The 1,3-DC between nitrones and alkenes is a well-known transformation leading to isoxazolidines. The reaction mechanism is affected by the electrostatic interaction between the nitrone and the alkenes. Alkyl substituents are electron-donating groups (EDG) and increase the electron density of the double bond through an inductive donating effect. To gain a further insight, we have also considered an electron-attractor group (EWG) as a substituent: an ester group. This effect is to increase the negative charge on carbon C_α_ depleting the electron density over the double bond. Another possible effect is the polarization of the alkene double bond given by the high dipole moment on the nitrone.

**Scheme 6:**
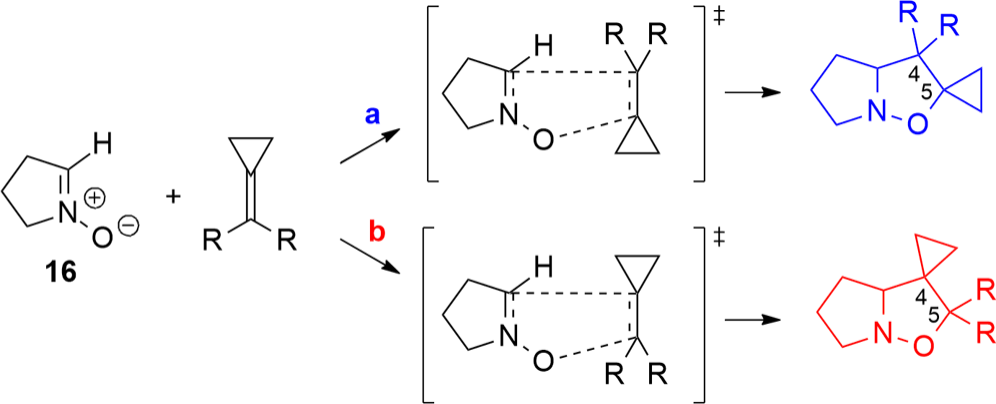
Transition States Originated from Different Paths **a** and **b** Leading to Regioisomeric Cycloadducts

The regioselectivity of the 1,3-DCs, i.e., the relative orientation of the substituted alkene to the nitrone in the TS, is influenced by the effect of the substituents on the dipolarophile. For this reason, we have considered two alkene orientations to explain the different selectivities for the studied reactions: the orientation labeled **a** leads to the product with the cyclopropane (or the cycloalkane) in position 5; label **b** refers to the orientation of the alkenes leading to the regioisomer spiro-fused in 4 (Scheme 6).

### Cycloaddition with Isobutene (**7**)

The 1,3-DC of a nitrone with isobutene (**7**) experimentally gives one single regioisomer, **9**, i.e., the 5,5-dimethyl-substituted isoxazolidine (see structure 8 in 2).

**Scheme 7:**
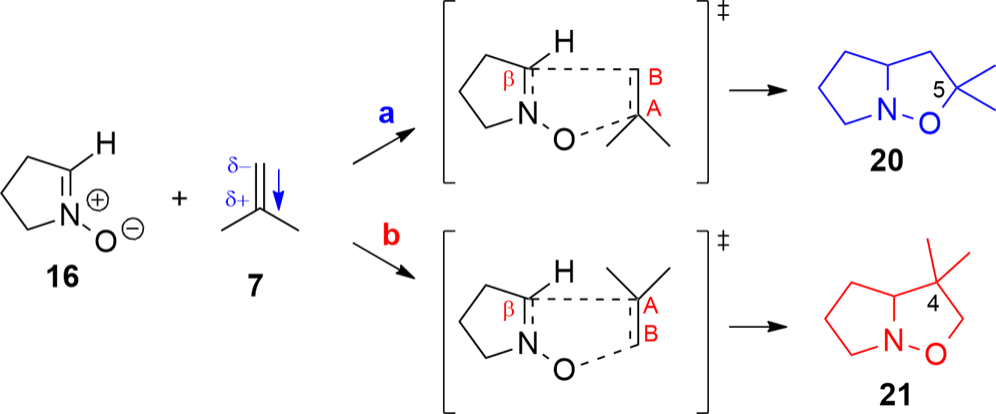
1,3-DC between Isobutene **7** and Nitrone **16**; Orientation **a** Leads to the 5,5-Dimethylisoxazolidine **20**, and Orientation **b** Leads to the 4,4-Dimethylisoxazolidine **21**; the Dipole Moment on **7** is Oriented toward the Dimethyl Group

The reaction paths obtained by the computational analysis on regioisomers **20** and **21** (Scheme 7) suggest an explanation for such regioselectivity. The path **a** of the 1,3-DC with the isobutene **7** leads to the formation of isoxazolidine **20** substituted with two methyl groups on position 5. This arrangement of the reagents has a lower activation energy (Δ*E*_TS_(**a** – **b**) = −18.0 kJ/mol), and the product **20** is more stable than isoxazolidine **21** (path **b**) (Table S8 in the Supporting Information).

The analysis of ESP charges has evidenced that the two methyl groups determine a positive charge on the C_A_ carbon (0.469) and a charge transfer toward the C_B_ (−0.737) of the isobutene (Scheme 7, Table S9 in the Supporting Information). This charge distribution on the isobutene corresponds to the dipole moment of 0.59 D, which lies on the C_A_–C_B_ bond and is oriented toward the EDG groups. In the prereactive minimum, the nitrone dipole further increases the polarization of isobutene, as evidenced by the charges of the carbons C_A_ and C_B_ reported in Table S16 in the Supporting Information. The reaction mechanism is favored by the **a**-arrangement of the reactants because the carbon C_A_ involved in the attack of the nitrone oxygen is positive (0.506). The polarization effect given by the nitrone in the **b** orientation determines instead a negative charge on C_B_ (−0.748) that makes the attack of nitrone oxygen less favored (Scheme 7). Therefore, the charge distribution and orientation of the alkene’s dipole moment justify the kinetic ratio calculated at the experimental temperature with Eyring’s equation (*k*_**a**/**b**_ = 114.4 at 350 K) and indicate the 5,5-disubstituted isoxazolidine **20** as the favorite product of the 1,3-DC.

### Cycloadditions with Methylenecyclopropane (MCP, **2**) and Methylenecyclobutane (MCB, **14**)

**Scheme 8:**
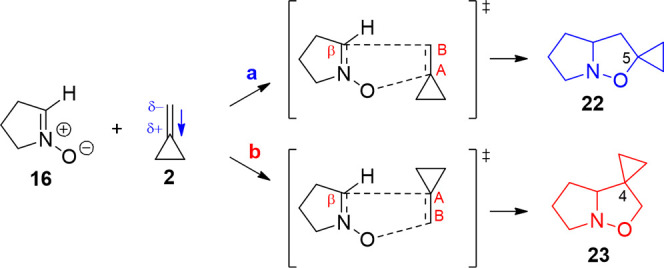
1,3-DC between MCP (2) and Nitrone **16**; Orientation **a** Leads to Isoxazolidine **22**, and Orientation **b** Leads to Isoxazolidine **23**; the Dipole Moment on **2** is Oriented toward the Cyclopropane

The comparison of cycloaddition of nitrone 11 with methylenecyclopropane (**2**, MCP) and methylenecyclobutane (**14**, MCB) is rather interesting as the former leads experimentally to the formation of an approximately 65:35 mixture of regioisomers, where the majority is the 5-spiro-fused isoxazolidine **12**, whereas MCB affords exclusively the 5-spiro-fused isoxazolidine **15** (Schemes 3 and 4). At a first glance, this difference is surprising since it contrasts with the apparent similarity of the two dipolarophiles. The computational analysis, indeed, infers an explanation for this different reactivity. The difference in activation energy between the TS of the two chosen orientations (**a**, **b**) of the MCP is moderate (Δ*E*_TS_(**a** – **b**) = −7.7 kJ/mol, Table S10 in the Supporting Information). Orientation **a** (Scheme 8) is favored and leads to 5-spirocyclopropane isoxazolidine **22**, which is also more stable than the isomeric 4-spirocyclopropane **23** obtained with the **b**-oriented MCP (Δ*E*(**a** – **b**) = −10.4 kJ/mol).

**Scheme 9:**
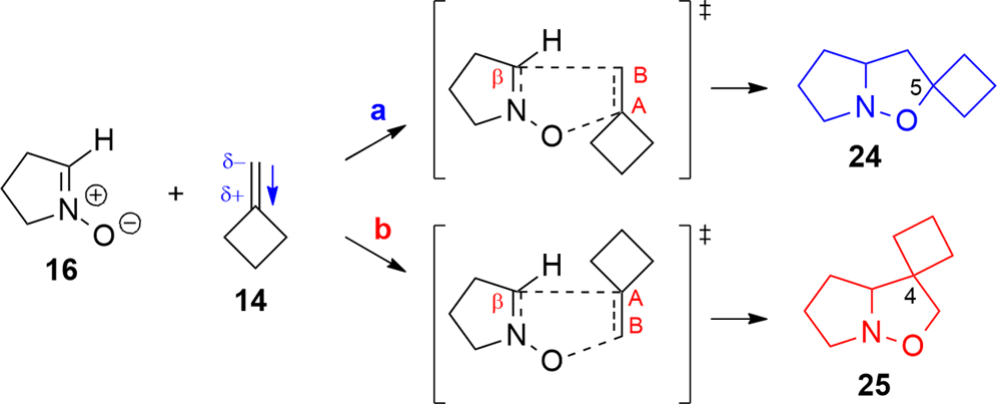
1,3-DC between MCB **14** and Nitrone **16**; Orientation **a** Leads to Isoxazolidine **24**, and Orientation **b** Leads to Isoxazolidine **25**; the Dipole Moment on the Alkene is Oriented toward the Cyclobutane

The difference in activation energy for the two chosen orientations of MCB is higher than in the previous case (Δ*E*_TS_(**a** – **b**) = −15.7 kJ/mol, Table S10 in the Supporting Information). Indeed, orientation **a** is favorable and leads to the more stable product with the spirocyclobutane in position 5 (**24**) (Δ*E*(**a** – **b**) = −23.6 kJ/mol) (Scheme 9). The inductive effect on the cyclopropane determines a carbon with a positive charge (C_A_) and one with a negative charge (C_B_, Table S11 in the Supporting Information). As a proof of this charge distribution on the double bond, the dipole moment on the MCP (0.455 D) is oriented toward the cyclopropane.

In the prereactive minima, the polarization of the MCP is increased by the nitrone dipole according to the orientation (Table S11 in the Supporting Information). The two sp2 carbons (C_A_, C_β_), as acceptors of electron density, are involved in the bonding at the transition state (Scheme 8). Therefore, the positive charge on C_A_ (0.147) and the less negative charge on C_β_ (−0.101) in the **a** arrangement favor the reaction outcome (Table S11 in the Supporting Information).

The inductive effect of the cyclobutane on the double bond of the MCB (Table S11 in the Supporting Information) is greater if compared to the cyclopropane one. Indeed, the dipole moment of the double bond in MCB (0.612 D) lies on the double bond axis and is oriented toward the cyclobutane substituent. The dipole moment of the nitrone increases the polarization of the dipolarophile. The arrangement of the reagents in orientation **a** determines a positive charge on alkene carbon C_A_ (0.237) and a less negative charge on the nitrone carbon C_β_ (−0.143), favoring the cycloaddition mechanism (Table S11 in the Supporting Information; see also Scheme 9). Therefore, the computational results for the 1,3-DC of MCP explain the experimental regioselectivity, i.e., the obtainment of two regioisomers with a prevalence of the product deriving from orientation **a**. Indeed, the two orientations do not differ greatly in terms of activation energy, neither on product stability. The ratio between rate constants obtained for the two orientations (*k*_**a**/**b**_ = 5) (Table S12 in the Supporting Information) suggests a reduction of the kinetic control of the regioselectivity in this cycloaddition. However, orientation **a** provides a relatively minor activation energy (Δ*E*_TS_(**a** – **b**) = −7.7 kJ/mol) and the most stable isoxazolidine (Δ*E*(**a** – **b**) = −10.4 kJ/mol). Also, the charge distribution on the alkene **2** in the **a** arrangement suggests that the isoxazolidine **22** with the spirocyclopropane substituent in position 5 is the favored product (Table S11 in the Supporting Information). In the reaction with MCB instead, the ratio between rate constants obtained for the two orientations (*k*_**a**/**b**_= 117) and the different stabilities of the two isoxazolidines indicate a kinetic control of the reaction. The analysis of the charge distribution on the double bond of the MCB confirms that the 5-spirocyclobutane isoxazolidine **24**, obtained with **a** arrangement, is the highly favored product (Table S12 in the Supporting Information). The different reactivities of MCP and MCB can be therefore explained by the kinetic and electrostatic behaviors in the two reactions. In the 1,3-DC with MCP, the lack of regioselectivity is due to a lower EDG effect of the substituent that leads to the formation of both **22** and **23** products according to the experimental ratio (**a** > **b**).^16,17^ Instead, the electronic effect of the cyclobutane determines a charge distribution over the alkene that is conducive to the formation of the isoxazolidine **24** substituted with the spirocyclobutane on position 5.

### Cycloadditions with Isopropylidenecyclopropane (ICP, **17**) and Isopropylidenecyclobutane (ICB, **18**)

The 1,3-DC between ICP and nitrones experimentally gives one single regioisomer featuring the methyl groups on C-5 of the isoxazolidine ring.

**Scheme 10:**
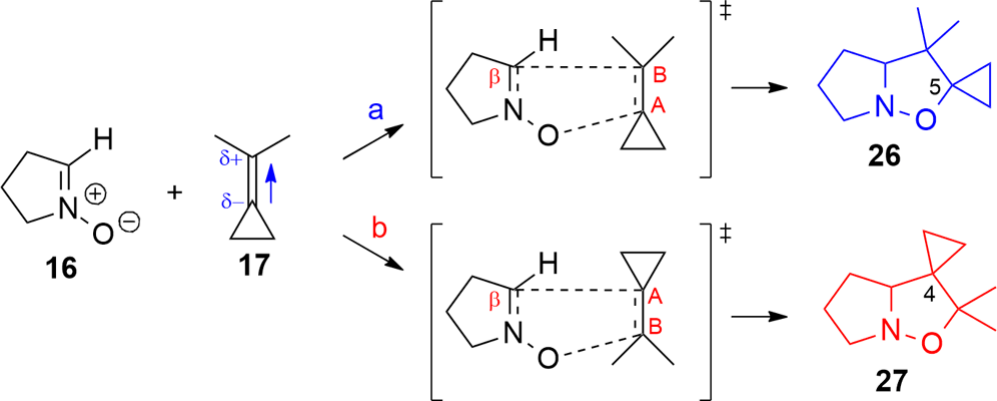
1,3-DC between ICP and Nitrone **16**; Orientation **a** Leads to Isomer **26**, and Orientation **b** Leads to Isomer **27**; the Dipole Moment on the Alkene is Oriented toward the Dimethyl Group

This complete regioselectivity can be explained by the different reactivity of the ICP (**17**) in the two studied orientations. Indeed, the computed cycloaddition between the ICP and nitrone **16** shows two reaction paths that are rather different, according to the orientation chosen: the proposed orientation **a** (Scheme 10) leads to the isoxazolidine **26** spiro-fused at C-5, and orientation **b** leads to the isomer **27**.

The arrangement of ICP proposed in orientation **a** has the highest activation energy of the studied reactions (99 vs 40–50 kJ/mol). The arrangement of the **b**-oriented ICP determines a sensibly lower activation energy (Δ*E*_TS_(**a** – **b**) = 61.5 kJ/mol) and leads to the more stable isoxazolidine **27**, 12.540 kJ/mol (Table S13 in the Supporting Information). This difference in activation energy and the kinetic ratio (*k*_**a**/**b**_ = 4.6 × 10^–11^) indicates a kinetic control of the reaction leading to the formation of one product, i.e., isomer 27 (Table S15 in the Supporting Information). All substituents of ICP are EDG groups, the dipole moment of the molecule is quite small (0.167 D) compared to the values of the other alkenes. The dipole lies on the double-bond axis and is oriented toward the dimethyl group, which means that the dimethyl provides the bigger EDG contribution to the alkene favoring the formation of **27**. Indeed, in orientation **b**, the ICP carbon involved in the intermolecular O–C bond formation, has a small positive charge (0.009), whereas, in the other orientation **a**, the carbon belonging to the cyclopropane ring (C_A_) experiences a negative charge (−0.020) (Table S14 in the Supporting Information).

From these data, it can be concluded that the cycloaddition between nitrone **16** and ICP **17** proceeds through the **b**-oriented TS, which is favored from a kinetic point of view and leads to the formation of the more stable product **27**. The ratio of kinetic constants between the orientations **a** and **b** (*k*_**a**/**b**_ = 10^–10^) (Table S15 in the Supporting Information) is much lower than 1, confirming that orientation **b** is kinetically much more favored. These results therefore explain the formation of isoxazolidine **27** as the exclusive product. Experimental data for the reaction of nitrones with the ICB (**18**) are not available, but this dipolarophile has been studied to evaluate the electronic effect of the two different cycloalkanes in the presence of the dimethyl group.

**Scheme 11:**
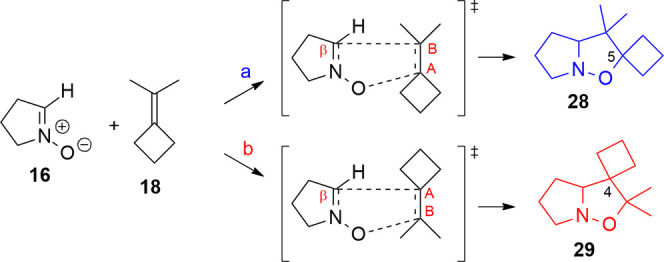
1,3-DC between ICB and Nitrone **16**; Orientation **a** Leads to Isoxazolidine **28**, and Orientation **b** Leads to Isomer **29**; the Dipole Moment of **18** is Close to Zero

**Scheme 12:**
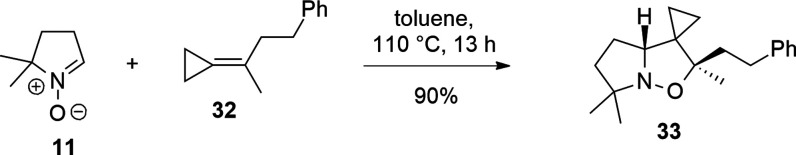
1,3-DC of Nitrone **11** with **32**

The reaction shows lower differences according to the chosen orientation of the alkene. The difference in activation energy for the two orientations of ICB with nitrone 16 is much lower (Δ*E*_TS_(**a** – **b**) = 3.8 kJ/mol) than the previous case: orientation **a** (Scheme 11) leads to the 5-spirocyclobutane-4,4-dimethylisoxazolidine 28, while orientation **b** leads to the more stable 5,5-dimethyl-4-spirocyclobutane isoxazolidine **29** (Δ*E*(**a** – **b**) = 15.8 kJ/mol) (Table S13 in the Supporting Information). As seen in the previous case, all substituents of the alkene are EDG groups: the value of the dipole moment (0.011 D) is close to the accuracy of the method, so no consideration can be made about the dipole orientation. However, the polarization induced by nitrone dipole and the EDG effect of the substituents promotes a positive charge on carbon C_B_ promoting the formation of isomer **29** (Table S14 in the Supporting Information). Indeed, the kinetic ratio of the reaction (*k*_**a**/**b**_ = 6 × 10^–2^) suggests that path **b** is slightly kinetically favored due to a better charge distribution over the reagents. Altogether, these data indicate that isoxazolidine **29** is only slightly favored (Table S15 in the Supporting Information). The compared assessment of reactions of ICP and ICB with nitrone **16** and their full computational analysis has brought about a sharp difference of behavior of a cyclopropylidene compared to a cyclobutylidene group. Indeed, for the ICB, the electronic effects of the dimethyl and cyclobutane are similar, as shown by the dipole moment value. Instead, the charges on the sp2 carbons (C_A_, C_B_) on the alkene **17** are determined by the stronger EDG effect of the two methyl groups compared to the cyclopropane; therefore, path **b** results in a more favorable charge distribution leading to isoxazolidine **27** (*k*_**a**/**b**_ = 6 × 10^–10^). Indeed, the cycloaddition of nitrone **11** with **32**, a dipolarophile analogous to ICB, experimentally gives only regioisomer **33**, originating from **b** orientation like **27**, in excellent yield (Scheme 12).^36^

### Cycloaddition with Cyclobutylidenecyclopropane (CBCP, **19**)

The computational study of the reaction of nitrone **16** with CBCP gives a further confirmation of the existence of this cyclopropylidene effect experimentally observed.

Experimentally, 1,3-DC of nitrone **34** with CBCP gave exclusively isoxazolidine **35** featuring the cyclopropane ring on position 4 of the isoxazolidine ring (**13**).^37^

The difference in activation energy (Δ*E*_TS_(**a** – **b**) = −6.9 kJ/mol) is in favor of the **b**-oriented alkene, which leads to the isoxazolidine substituted with the cyclobutane on position 5 (isomer **31**) that is also thermodynamically more stable (−9.0 kJ/mol) than the other possible product (isomer **30**) deriving from orientation **a** (Table S16 in the Supporting Information).

**Scheme 13:**
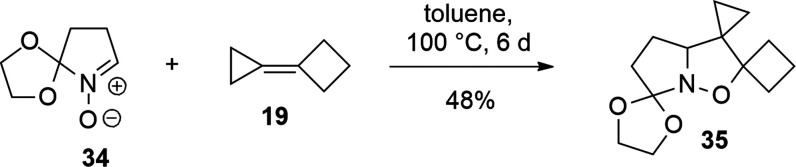
1,3-DC of Nitrone **34** with CBCP (**19**)

**Scheme 14:**
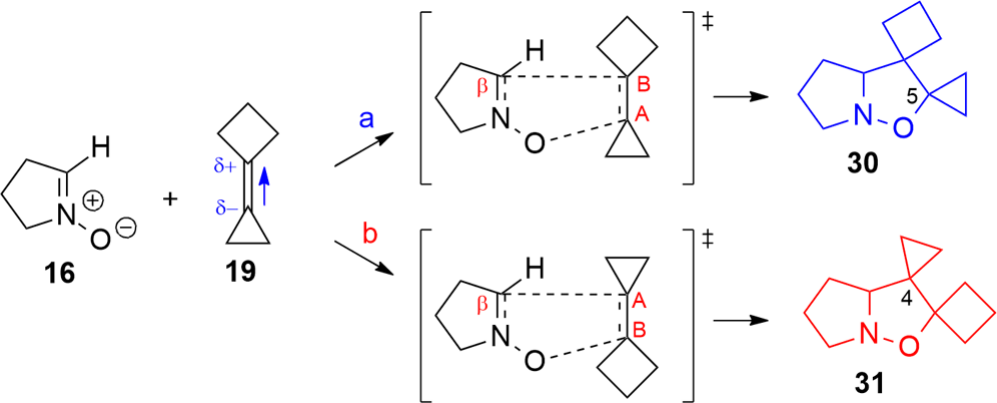
1,3-DC of **19** with Nitrone **16**; Orientation **a** Leads to Isoxazolidine 30, and Orientation **b** Leads to Isomer **31**; the Dipole Moment on the Alkene is Oriented toward the Cyclobutane

The alkene dipole moment is quite small (0.168 D), since all substituents are EDG groups, and is oriented toward the cyclobutane, due to its greater inductive effect on the molecule. In the **a** arrangement, the carbon C_A_ is involved in the formation of C–O bond, while in orientation **b**, the carbon involved is C_B_. As in the previous cases, the nitrone dipole polarizes the alkene double bond, increasing the partial charges on the sp2 carbon atoms. The reaction mechanism is therefore favored in orientation **b** by the positive charge on carbon C_B_ (0.125, Table S17 in the Supporting Information). These results and the kinetic ratio of the reaction (*k*_**a**/**b**_ = 0.1) indicate that isoxazolidine **31** is the favored product in complete agreement with the experimental data (Scheme 13 and Scheme 14).

### Cycloaddition with Cyclopropylideneacetate **36**

We have also investigated the effect of an electron-attractor group (EWG), i.e., an ester group, combined with the cyclopropane.

**Scheme 15:**
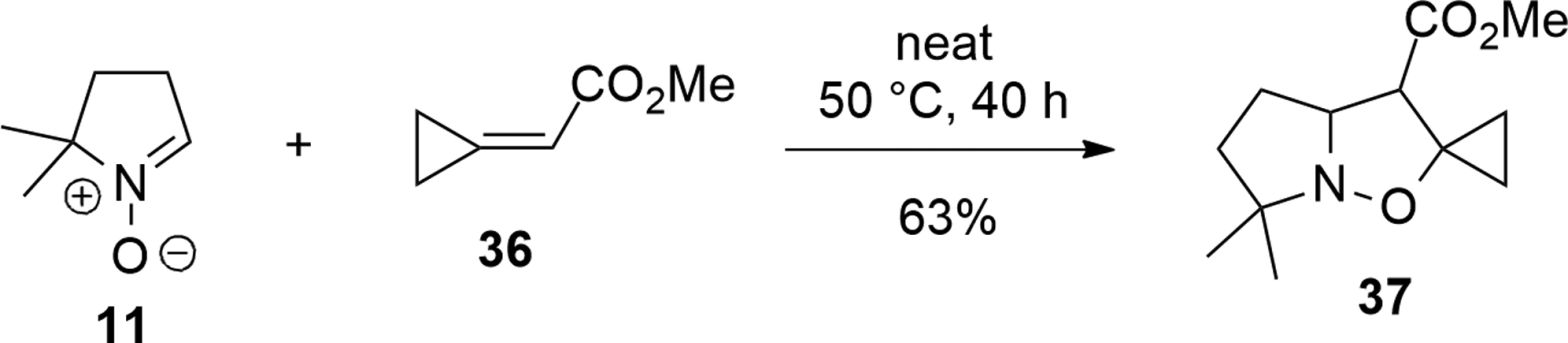
1,3-DC of Cyclopropylideneacetate **36** with Nitrone **11**

1,3-DC of nitrones with **36** gives experimentally only isoxazolidines substituted with the spirocyclopropane on position 5, as shown in the example of Scheme 15. The outcome of the cycloaddition is therefore inverted with respect to the results shown previously. This depends on the electron-withdrawing character of the CO_2_Me substituent.

**Scheme 16:**
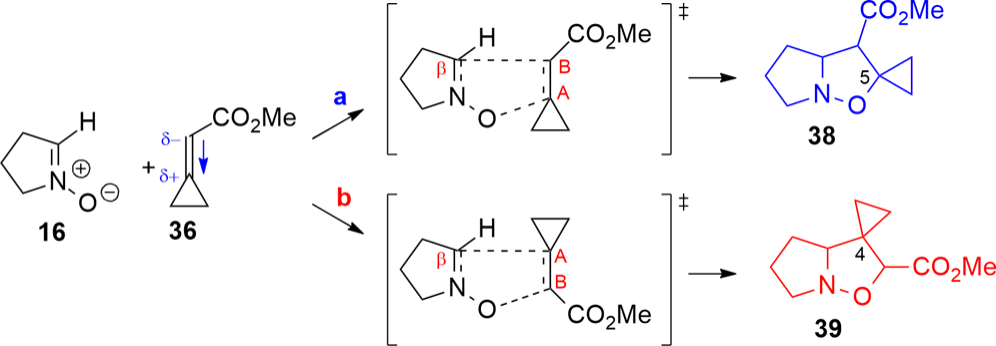
1,3-DC between Cyclopropylideneacetate **36** and Nitrone **16**; Orientation **a** Leads to Isoxazolidine **38**, and Orientation **b** Leads to Isoxazolidine **39**; the Dipole Moment on the Alkene is Oriented toward the Cyclopropane

The alkene is substituted on C_A_ with the cyclopropane, and on C_B_ with the carboxymethyl group; therefore, the carbon C_A_ is the β carbon compared to the carbonyl acetate (**16**). Both the ester and alkyl groups contribute to polarize the double bond, increasing the negative charge on C_B_ (−0.807) and consequently the positive charge on C_A_ (0.447, Table S19 in the Supporting Information). Indeed, the resulting dipole moment (3.064 D) is oriented toward the cyclopropane group and this value is higher compared to the previous cases due to the methyl carboxylate effect. In orientation **a**, the positive charge on carbon C_A_ enhances the attack on the nitrone oxygen (Scheme 16). Indeed, this arrangement of the reagents determines the lowest activation energy (Δ*E*(**a** – **b**) = −14.1 kJ/mol, Table S18 in the Supporting Information) and leads to the formation of 5-spirocyclopropane isoxazolidine **38**. The polarization of the double bond and the resultant negative charge on carbon C_B_ make **b** orientation less favored. Also, the nitrone dipole moment increases the polarization of the alkene double bond, favoring the charge distribution adopted in orientation **a**. Indeed, the kinetic constant rates (*k*_**a**/**b**_ = 123) obtained with the Eyring equation show that the isoxazolidine **38** is the kinetic product of the 1,3-DC, in agreement with the experimental data.

## Conclusions

The lack of regioselectivity in 1,3-DC of methylenecyclopropane (MCP, **2**), compared to the high regioselectivity of other 1,1-disubstituted alkenes, has been computationally investigated through DFT calculations, to rationalize the experimental finding. The computational analysis has been extended to tetrasubstituted alkenes and to cyclopropylideneacetate to substantiate the results. It has been observed that the electrostatic interactions play a fundamental role in the regioselectivity of these 1,3-DC reactions. In fact, the electronic structure of the substituents (EDG and EWG) causes polarization of the alkene double bond, which in turn favors a proper structural arrangement between the reactants and consequently controls the regioselectivity. The comparison of the reactions between nitrone **16** and 1,1-disubstituted alkenes isobutene (**7**) and MCB (**14**) shows that for similar substituents (dimethyl, cyclobutyl), the reaction outcome is essentially due to the electrostatic interaction (atomic charges and dipole moment), with a preferential formation of the 5,5-disubstituted isoxazolidine. In the case of MCP, where the electrostatic interaction is less marked, the difference in 5- and 4-spiro-fused isoxazolidine formation is lower, and consequently the kinetic constants of the two paths are similar (the kinetic constant ratio is only *k*_**a**/**b**_ = 5). The electronic structure analysis of 1,3-DC with the tetrasubstituted alkenes ICP (**17**), ICB (**18**), and CPCB (**19**) reveals a smaller value of the dipole moment because all of the substituents are alkyl groups, and the partial charges on the double bond are given by the subtraction of the substituent EDG effects. The alkenes ICP and CPCB feature both a cyclopropyl group on one end of the double bond, and on the other end, a geminal dimethyl group and a cyclobutyl ring, respectively. The comparison of these two dipolarophiles allows us to understand how the cycloaddition reaction is influenced by the alkene substituents with the largest EDG effect. In ICP, the EDG effect of the geminal dimethyl is stronger than the one of cyclopropyl: the *k*_**a**/**b**_ ratio shows that the reaction is completely shifted toward the formation of isomer **27** with methyl groups on C-5 (*k*_**a**/**b**_ = 6 × 10^–10^). A similar behavior, although with a lower weight, occurs in CPCB. In the case of 1,3-DC with ICB, the electrostatic contributions of the substituents are similar; thus, the polarization of the double bond is small but still enough to favor the formation of the isoxazolidine with methyl groups on C-5 (*k*_**a**/**b**_ = 6 × 10^–2^). The cyclopropylideneacetate **36** is the only example studied featuring a cyclopropyl ring and an EWG. In this case, the two electronic effects sum, as shown by the value and orientation of the alkene dipole moment. The contribution of the EWG is dominant and leads exclusively to the formation of product **39** (*k*_**a**/**b**_ = 123). Summing up, the electronic effect of the alkene substituents measured in this study determines a polarization of the alkene double bond favoring the relative orientation between the reactants leading to the observed regioselectivity of the cycloadditions. This study has allowed us to explain the role of a cyclopropylidene group (the cyclopropylidene effect) in inducing regioselectivity in nitrone 1,3-dipolar cycloadditions.

## Supporting Information Available

Comparison between ESP, CM5, and NPA population analysis (Tables S1–S7); energy variations and ESP charges for the reaction involving isobutene, respectively (Tables S8 and S9); energy variations, ESP charges, and transmission coefficients for the reaction involving methylenecyclopropane and methylenecyclobutane, respectively (Tables S10–S12); energy variations, ESP charges, and transmission coefficients for the reaction involving isopropylidenecyclopropane and isopropylidenecyclobutane, respectively (Tables S13–S15); energy variations and ESP charges for the reaction involving cyclobutylidenecyclopropane, respectively (Tables S16 and S17); and energy variations and ESP charges for the reaction involving cyclopropylideneacetate, respectively (Tables S18 and S19) (PDF)
