# Supplementary material for: “Cyclopropylidene Effect” in the 1,3-Dipolar Cycloaddition of Nitrones to Alkylidene Cyclopropanes: A Computational Rationalization
Source: J Phys Chem A. 2021 Apr 30;125(18):3892–9. doi: 10.1021/acs.jpca.1c02204 (PMC8279640; doi:10.1021/acs.jpca.1c02204)
Supplement: Supplementary file 1 — jp1c02204_si_001.pdf [file jp1c02204_si_001.pdf]

Supporting Information
The "Cyclopropylidene Effect" in the 1,3-Dipolar Cycloaddition of Nitrones to Alkylidene cyclopropanes. A Computational Rationalization

Lorenzo Briccolani-Bandini, Marco Pagliai,* Franca M. Cordero,* Alberto Brandi,* and Gianni Cardini*

Dipartimento di Chimica "Ugo Schiff", Università degli studi di Firenze, Via della Lastruccia 3-13, 50019 Sesto Fiorentino (Firenze), Italy

E-mail: marco.pagliai@unifi.it; franca.cordero@unifi.it; alberto.brandi@unifi.it; gianni.cardini@unifi.it

Table S1: The ESP, CM5, NPA charges have been reported for the sp2 carbons of the isobutene 7 (C_A, C_B) and for the interacting atoms of the nitrone 16 (C_β, N, O). The charge have been calculated for the isolated reactants (nitrone and isobutene).

	ESP	CM5	NPA
$C_{A(\mathbf{7})}$	0.469217	-0.021094	-0.00836
$C_{B(\mathbf{7})}$	-0.736784	-0.214182	-0.41187
$O_{A(\mathbf{16})}$	-0.566773	-0.302139	-0.53059
$N_{A(\mathbf{16})}$	0.441358	-0.115015	0.08629
$C_{\beta(\mathbf{16})}$	-0.230350	0.020439	-0.00385

Table S2: The ESP, CM5, NPA charges have been reported for the sp2 carbons of the isobutene 2 (C_A, C_B) and for the interacting atoms of the nitrone 16 (C_β, N, O). The charge have been calculated for the isolated reactants (nitrone and isobutene).

	ESP	CM5	NPA
$C_{A(\mathbf{2})}$	0.281712	-0.037240	-0.02053
$C_{B(\mathbf{2})}$	-0.620743	-0.197492	-0.39213
$O_{A(\mathbf{16})}$	-0.566773	-0.302139	-0.53059
$N_{A(\mathbf{16})}$	0.441358	-0.115015	0.08629
$C_{\beta(\mathbf{16})}$	-0.230350	0.020439	-0.00385

Table S3: The ESP, CM5, NPA charges have been reported for the sp2 carbons of the isobutene 14 (C_A, C_B) and for the interacting atoms of the nitrone 16 (C_β, N, O). The charge have been calculated for the isolated reactants (nitrone and isobutene).

	ESP	CM5	NPA
$C_{A(\mathbf{14})}$	0.271190	-0.022674	0.00339
$C_{B(\mathbf{14})}$	-0.698235	-0.214625	-0.41875
$O_{A(\mathbf{16})}$	-0.566773	-0.302139	-0.53059
$N_{A(\mathbf{16})}$	0.441358	-0.115015	0.08629
$C_{\beta(\mathbf{16})}$	-0.230350	0.020439	-0.00385

Table S4: The ESP, CM5, NPA charges have been reported for the sp2 carbons of the isobutene 17 (C_A, C_B) and for the interacting atoms of the nitrone 16 (C_β, N, O). The charge have been calculated for the isolated reactants (nitrone and isobutene).

	ESP	CM5	NPA
$C_{A(\mathbf{17})}$	0.100976	-0.04619	-0.04619
$C_{B(\mathbf{17})}$	0.159697	-0.02542	-0.02542
$O_{A(\mathbf{16})}$	-0.566773	-0.302139	-0.53059
$N_{A(\mathbf{16})}$	0.441358	-0.115015	0.08629
$C_{\beta(\mathbf{16})}$	-0.230350	0.020439	-0.00385

Table S5: The ESP, CM5, NPA charges have been reported for the sp2 carbons of the isobutene 18 (C_A, C_B) and for the interacting atoms of the nitrone 16 (C_β, N, O). The charge have been calculated for the isolated reactants (nitrone and isobutene).

	ESP	CM5	NPA
$C_{A(\mathbf{18})}$	-0.039164	-0.044737	-0.04368
$C_{B(\mathbf{18})}$	-0.037420	0.103927	-0.02125
$O_{A(\mathbf{16})}$	-0.566773	-0.302139	-0.53059
$N_{A(\mathbf{16})}$	0.441358	-0.115015	0.08629
$C_{\beta(\mathbf{16})}$	-0.230350	0.020439	-0.00385

Table S6: The ESP, CM5, NPA charges have been reported for the sp2 carbons of the isobutene 19 (C_A, C_B) and for the interacting atoms of the nitrone 16 (C_β, N, O). The charge have been calculated for the isolated reactants (nitrone and isobutene).

	ESP	CM5	NPA
$C_{A(\mathbf{19})}$	0.086844	-0.026871	-0.01267
$C_{B(\mathbf{19})}$	-0.003760	-0.054891	-0.05332
$O_{A(\mathbf{16})}$	-0.566773	-0.302139	-0.53059
$N_{A(\mathbf{16})}$	0.441358	-0.115015	0.08629
$C_{\beta(\mathbf{16})}$	-0.230350	0.020439	-0.00385

Table S7: The ESP, CM5, NPA charges have been reported for the sp2 carbons of the isobutene 36 (C_A, C_B) and for the interacting atoms of the nitrone 16 (C_β, N, O). The charge have been calculated for the isolated reactants (nitrone and isobutene).

	ESP	CM5	NPA
$C_{A(\mathbf{36})}$	0.447448	-0.005028	0.06265
$C_{B(\mathbf{36})}$	-0.807355	-0.108089	-0.31767
$O_{A(\mathbf{16})}$	-0.566773	-0.302139	-0.53059
$N_{A(\mathbf{16})}$	0.441358	-0.115015	0.08629
$C_{\beta(\mathbf{16})}$	-0.230350	0.020439	-0.00385

Table S8: The $\delta s.p.$ represents the difference in energy of interacting reactants and the energy of the isolated isobutene 7 and 16 for each stationary point of the reaction ($\delta s.p. = E_{s.p.} - E_{\mathbf{7}} - E_{\mathbf{16}}$). The ΔE represents the difference in energy between the chosen orientation (Path a and b) of the isobutene. The differences in energy are expressed in kJ/mol.

$\delta s.p.$	$E_{Path\mathbf{a}}$	$E_{Path\mathbf{b}}$	$\Delta E_{\mathbf{a-b}}$
δr	-32.8	-30.5	-2.3
δts	35.4	53.4	-18.0
δp	-143.6	-118.0	-25.6

Table S9: The ESP charges have been reported for the sp2 carbons of the isobutene 7 (C_A, C_B) and for the interacting atoms of the nitrone 16 (C_β, N, O). The ESP charge have been calculated for the isolated reactants (nitrone and isobutene) and for the interacting species in the pre-reactive minimum for the chosen orientations a and b.

Atoms	Isolated	Path **a**	Path **b**
$C_{A(\mathbf{7})}$	0.469	0.506	0.542
$C_{B(\mathbf{7})}$	-0.737	-0.940	-0.748
$O_{A(\mathbf{16})}$	-0.567	-0.570	-0.560
$N_{A(\mathbf{16})}$	0.441	0.399	0.382
$C_{\beta(\mathbf{16})}$	-0.230	-0.138	-0.185

Table S10: The $\delta s.p.$ represents the difference in energy of interacting reactants and the energy of the isolated alkene 2, 14 and 16 for each stationary point of the reaction ($\delta s.p. = E_{s.p.} - E_{\mathbf{nitr.}} - E_{\mathbf{alk.}}$); while the ΔE represents the difference in energy between the two dipolarophile possible orientations. Difference in energy expressed in kJ/mol.

$\delta s.p.$	$E_{\mathbf{2Patha}}$	$E_{\mathbf{2Pathb}}$	$\Delta E_{\mathbf{a-b}}$	$E_{\mathbf{14Patha}}$	$E_{\mathbf{14Pathb}}$	$\Delta E_{\mathbf{a-b}}$
δr	-27.2	-23.7	-3.5	-33.8	-37.9	4.1
δts	28.7	36.5	-7.8	26.5	42.3	-15.8
δp	-172.7	-162.3	-10.4	-158.6	-135.0	-23.6

Table S11: ESP charges in the pre-reactive minima for dipolarophiles MCP and MCB (C_A, C_B) and nitrone atoms (O, N, C_β) for the isolated molecules and the chosen orientations a and b.

Atoms	MCP (**2**)			MCB (**14**)		
	Isolated	Path **a**	Path **b**	Isolated	Path **a**	Path **b**
C_A	0.282	0.147	0.266	0.271	0.237	0.236
C_B	-0.621	-0.512	-0.591	-0.698	-0.675	-0.695
O_{16}	-0.567	-0.533	-0.556	-0.567	-0.553	-0.589
N_{16}	0.441	0.401	0.367	0.441	0.389	0.447
$C_{\beta(16)}$	-0.230	-0.101	-0.151	-0.230	-0.143	-0.265

Table S12: The transmission coefficient (k) has been calculated using the Eyring transition state theory and describes the changing in the rate of a chemical reaction against the experimental temperature.

Dipolarophile	Texp. (K)	k_{Eyring} Path **a**	k_{Eyring} Path **b**	$k_{\mathbf{a/b}}$
**2**	330	$1\,e^{11}$	$2\,e^{10}$	4.8
**14**	370	$1\,e^{12}$	$1\,e^{10}$	$1\,e^{2}$

Table S13: The $\delta s.p.$ represents the difference in energy of interacting reactants and the energy of the isolated alkene 17, 18 and 16 for each stationary point of the reaction ($\delta s.p. = E_{s.p.} - E_{\mathbf{nitr.}} - E_{\mathbf{alk.}}$); while the ΔE represents the difference in energy between the two dipolarophile possible orientations. Difference in energy expressed in kJ/mol.

$\delta s.p.$	$E_{\mathbf{17Patha}}$	$E_{\mathbf{17Pathb}}$	$\Delta E_{\mathbf{a-b}}$	$E_{\mathbf{18Patha}}$	$E_{\mathbf{18Pathb}}$	$\Delta E_{\mathbf{a-b}}$
δr	-42.3	-43.6	1.3	-49.9	-52.8	2.9
δts	85.8	24.3	61.5	33.1	29.3	3.8
δp	-164.5	-177.1	12.1	-130.9	-146.7	15.8

Table S14: ESP charges in the pre-reactive minima for ICP and ICB (CA, CB) and nitrone (O, N, C_β) atoms for the isolated molecules and the chosen orientations a and b

Atoms	ICP (**17**)			ICB (**18**)		
	Isolated	Path **a**	Path **b**	Isolated	Path **a**	Path **b**
C_A	0.101	-0.020	0.218	-0.045	-0.045	-0.006
C_B	0.160	0.263	0.009	0.104	0.195	0.125
O_{16}	-0.567	-0.533	-0.548	-0.567	-0.552	-0.539
N_{16}	0.441	0.380	0.364	0.441	0.397	0.343
$C_{\beta(16)}$	-0.230	-0.112	-0.221	-0.230	-0.188	-0.222

Table S15: The transmission coefficient (k_{Eyring}(1/M s)) has been calculated using the Eyring transition state theory and describes the changing in the rate of a chemical reaction against the experimental temperature.

Dipolarophile	Texp. (K)	k_{Eyring} Path **a**	k_{Eyring} Path **b**	$k_{\mathbf{a/b}}$
**17**	380	$5\,e^{2}$	$6\,e^{11}$	$6\,e^{-9}$
**18**	330	$4\,e^{7}$	$7\,e^{8}$	$5\,e^{-2}$

Table S16: The $\delta s.p.$ represents the difference in energy of interacting reactants and the energy of the isolated alkene 19 and 16 for each stationary point of the reaction ($\delta s.p. = E_{s.p.} - E_{\mathbf{nitr.}} - E_{\mathbf{alk.}}$); while the ΔE represents the difference in energy between the two dipolarophile possible orientations. Difference in energy expressed in kJ/mol.

$\delta s.p.$	$E_{Path\mathbf{a}}$	$E_{Path\mathbf{b}}$	$\Delta E_{\mathbf{a-b}}$
δr	-40.4	-45.2	4.8
δts	19.5	26.4	-6.9
δp	-190.9	-181.9	-9.0

Table S17: ESP charges in the pre-reactive minima for CBCP(19) (C_A, C_B) and nitrone 16 (C_β, N, O) atoms for the isolated molecules at the chosen orientations a and b.

Atoms	Isolated	Path **a**	Path **b**
$C_{A(\mathbf{19})}$	-0.004	-0.097	-0.006
$C_{B(\mathbf{19})}$	0.087	0.172	0.125
$O_{(\mathbf{16})}$	-0.567	-0.512	-0.539
$N_{(\mathbf{16})}$	0.441	0.323	0.343
$C_{\beta(\mathbf{16})}$	-0.230	-0.248	-0.222

Table S18: The $\delta s.p.$ represents the difference in energy of interacting reactants and the energy of the isolated alkene 36 and 16 for each stationary point of the reaction ($\delta s.p. = E_{s.p.} - E_{\mathbf{nitr.}} - E_{\mathbf{alk.}}$); while the ΔE represents the difference in energy between the two dipolarophile possible orientations. Difference in energy expressed in kJ/mol.

$\delta s.p.$	$E_{Path\mathbf{a}}$	$E_{Path\mathbf{b}}$	$\Delta E_{\mathbf{a-b}}$
δr	-40.8	-34.3	-6.5
δts	0.6	14.7	-14.1
δp	-149.6	-146.9	-2.7

Table S19: The ESP charges in the pre-reactive minima for dipolarophile 36 (C_A, C_B) and nitrone 16 (C_β, N, O) atoms for the isolated molecules at the chosen orientations a and b.

Atoms	Isolated	Path **a**	Path **b**
$C_{A(\mathbf{36})}$	0.447	0.443	0.472
$C_{B(\mathbf{36})}$	-0.807	-0.818	-0.848
$O_{A(\mathbf{16})}$	-0.567	-0.541	-0.571
$N_{A(\mathbf{16})}$	0.441	0.348	0.418
$C_{\beta(\mathbf{16})}$	-0.230	-0.236	0.225
